# Risk Factors for HIV and Unprotected Anal Intercourse among Men Who Have Sex with Men (MSM) in Almaty, Kazakhstan

**DOI:** 10.1371/journal.pone.0043071

**Published:** 2012-08-24

**Authors:** Mark Berry, Andrea L. Wirtz, Assel Janayeva, Valentina Ragoza, Assel Terlikbayeva, Bauyrzhan Amirov, Stefan Baral, Chris Beyrer

**Affiliations:** 1 Johns Hopkins Bloomberg School of Public Health, Department of Epidemiology, Baltimore, Maryland, United States of America; 2 Amulet Public Organization, Almaty, Kazakhstan; 3 Global Health Research Center of Central Asia, Almaty, Kazakhstan; UCL Institute of Child Health, University College London, United Kingdom

## Abstract

**Introduction:**

Men who have sex with men (MSM) are at high risk for HIV infection. MSM in Central Asia, however, are not adequately studied to assess their risk of HIV transmission. Methods: This study used respondent driven sampling methods to recruit 400 MSM in Almaty, the largest city in Kazakhstan, into a cross-sectional study. Participation involved a one-time interviewer-administered questionnaire and rapid HIV screening test. Prevalence data were adjusted for respondent network size and recruitment patterns. Multivariate logistic regression was used to investigate the association between HIV and selected risk factors, and unprotected anal intercourse (UAI) and selected risk factors.

**Results:**

After respondent driven sampling (RDS) weighted analysis, 20.2% of MSM were HIV-positive, and 69.0% had unprotected sex with at least one male partner in the last 12 months. Regression analysis showed that HIV infection was associated with unprotected receptive anal sex (AOR: 2.00; 95% CI: 1.04–3.84). Having unprotected anal intercourse with male partners, a measure of HIV risk behaviors, was associated with being single (AOR: 0.38; 95% CI: 0.23–0.64); very difficult access to lubricants (AOR: 11.08; 95% CI: 4.93–24.91); STI symptoms (AOR: 3.45; 95% CI: 1.42–8.40); transactional sex (AOR: 3.21; 95% CI: 1.66–6.22); and non-injection drug use (AOR: 3.10; 95% CI: 1.51–6.36).

**Conclusions:**

This study found a high HIV prevalence among MSM in Almaty, and a population of MSM engaging in multiple high-risk behavior in Almaty. Greater access to HIV education and prevention interventions is needed to limit the HIV epidemic among MSM in Almaty.

## Introduction

Studies conducted around the world consistently find that men who have sex with men (MSM) tend to be underserved, and have much higher risk of HIV acquisition than the heterosexual population, even in countries with generalized epidemics [1]. The disparity in HIV prevalence comparing MSM and the general population is found in many countries. A metanalysis of data from 38 countries found that MSM had a 19.3 times higher odds of HIV infection compared with the overall HIV prevalence [1]. The majority of data regarding the relative contribution of MSM to the HIV epidemic as a whole has been generated in high income countries, including the U.S., Australia, and Western European countries. However, there is now a significant body of evidence demonstrating that MSM are also at high risk for infection in low and middle income countries [1].

The Central Asian region has an expanding epidemic among injecting drug users, but much less is known about HIV among MSM [2]. Central Asia has one of the most glaring gaps of research on HIV and health risks among MSM [3]. Research has indicated that the HIV epidemic among countries in the former Soviet Union (which includes Central Asia) is escalating. This is mostly attributed to injecting drug use, and possibly partly in consequence to the social and economic upheaval caused by the disintegration of the Soviet Union [4], [5], while sexual transmission has recently risen as a mode of transmission in several countries [2]. In fact, Central Asia and Eastern Europe have some of the most rapidly escalating HIV epidemics in the world [2]. Up to 75% of the HIV infections in the region are among people who inject drugs. The HIV prevalence in Kazakhstan among injection drug users (IDU) is 4%, based on sentinel surveillance, and some cities are seeing outbreaks of HIV among IDU [6]. Infections are rising in other groups, including female sex workers and their clients, migrants and prisoners [6]. Although injecting drug use likely contributes to the majority of HIV transmission in Central Asia [6], there is a need to monitor HIV prevalence and risk factors in other most at-risk populations.

Epidemiologic assessments of HIV among these at-risk populations, including the MSM population, have received growing government attention in Kazakhstan in recent years, though there remains a dearth of independent scientific investigations. The WHO and UNAIDS reported 27 cases of HIV among MSM in Kazakhstan, Central Asia's largest country and largest economy, from 2002 to 2006 [7]. One study in Kazakhstan found no HIV cases, but sampled only 100 MSM [1]. Sentinel surveillance has been conducted in Kazakhstan as well asa convenience sample study of 450 men, both of which that found very low HIV prevalence, but high levels of HIV risk behaviors and poor knowledge of how HIV is transmitted [8]. UNAIDS estimated a 1% HIV prevalence among MSM in Kazakhstan in 2007, but a much higher (10.8%) prevalence among MSM in neighboring Uzbekistan [2]. A meta-analysis from Caceres et al. found that MSM population prevalence and risk factors for HIV among MSM suggested that between 45 and 52% of MSM in Central Asia participated in high-risk sex (unprotected anal sex or commercial sex) in the previous year [3]. The lack of consistency in the results of these surveillance efforts indicates a need for rigorous sampling techniques to understand the level of risk behavior and HIV prevalence of MSM in Kazakhstan.

Many studies of MSM in low- and middle-income countries rely on convenience samples, such as those conducted in the streets or STI clinics. In these samples, high-risk sub-populations such as male sex workers and male-to-female transgenders may be overrepresented [9]. Probability sampling techniques allow researchers to draw conclusions about the general population under study.

The goal of this study was to measure the HIV risk factors and HIV prevalence among MSM in Almaty, Kazakhstan's economic capital and largest city. This information is needed to inform the type and size of prevention and advocacy responses. While HIV was the primary outcome of interest, we anticipated that the inception of the HIV epidemic among MSM may be recent in Kazakhstan and, therefore, the relationship between HIV and traditional risk factors may not follow the expected patterns, because HIV may not yet exist in some sexual networks. Therefore, we also studied the association between selected risk factors and unprotected anal intercourse (UAI) with a male partner in the last 12 months, as UAI is a good indicator of sexual risk.

## Methods

### Setting, participants and study design

This cross-sectional study recruited MSM (defined for the purposes of this study as any man reporting oral or anal intercourse with another man in the last 12 months), 18 years or older, who were residing in Almaty at the time of enrollment. An Almaty-based lesbian, gay, bisexual and transgender (LGBT) non-governmental organization (NGO) provided experiential input to the design of the study and were responsible for participant recruitment and data collection.

Participants were recruited using respondent-driven sampling (RDS) methods [10]. RDS was selected over venue time sampling, as formative work identified too few (N = 2) venues for this approach to be feasible. RDS uses a peer-driven chain referral method, in which participants recruit a limited number of people from their social network. Traditional RDS methods are described in further details elsewhere [10]. Briefly, four “seeds,” who are members of the target community, were recruited by the Almaty-based NGO after participating in formative research activities. Each seed initiated the chain referrals and were provided with two coupons to recruit up to two MSM members from within the participant's social network. Each of those eligible recruits who complete the survey were, in turn, provided with two coupons to recruit MSM members of their social networks. Seeds and recruiters were paid the equivalent of $10 for participating in the study and $2.50 for each peer the participant recruited. Repeat participation was avoided by through eligibility assessment which asked if the potential participant had recently completed a survey for the implementing NGO and supported by staff recognition of duplicate participants. This process of referral continued until the sample size was reached; the sample size (400) of this study was calculated as the ability to detect a prevalence of unprotected anal intercourse of 25%, with adequate precision and a design effect of 2. RDS is a quasi-random sampling method, and analysts must statistically weight data for different probabilities of recruitment into the sample and for different patterns of social connections using a measure of network size and homophily (likeness) of recruiters to recruits for each variable of interest [11]. Ethical approval of the study was received from the Johns Hopkins Bloomberg School of Public Health Institutional Review Board and the Kazakhstan School of Public Health. Interviewers received oral consent from participants, to ensure their anonymity. The oral consent process was approved by both ethics committees. Interviewers recited the oral consent form, checked for understanding, and wrote his or her name on the form after receiving oral consent from participants.

### Measures and data collection

Participants answered an interviewer-administered paper-based questionnaire with approximately 100 variables on the following domains: demographics; sexual behavior with male and female partners; alcohol and drug use history; HIV prevention and testing experiences; health conditions; and human rights contexts. The questionnaire was adapted from other questionnaires used for other sociobehavioral assessments of MSM [12]. HIV knowledge was assessed by the number of questions participants correctly answered. These four questions included: “What type of sex puts you most at risk for HIV infection?”; “What type of anal sex puts you most at risk for HIV infection?”; “Can you get HIV from using a needle to inject drugs?”; “What is the safest kind of lubricant for anal sex?” Transactional sex with another man was assessed by asking participants if they had given or received food, drugs, money, or other items of value in exchange for sex in the last 12 months. Condom and lubricant access were assessed through a likert scale with response options ranging from “very easy” to “very difficult.”

A physician or nurse trained in HIV testing from the NGO assessed HIV-1 infection via fingerprick-acquired specimens collected and analyzed with Retrocheck HIV WB rapid immunochromatographic test (Qualpro Diagnostics, Goa, India), with 100% sensitivity and 99.8% sensitivity. Due to funding limitations, only one rapid test was performed; no confirmatory test was conducted and participants did not receive HIV results, due to the concern over false positive results. All participants were referred to the Almaty City AIDS Center for confirmatory testing. Sensitivity and specificity of the test was confirmed at the Johns Hopkins University serology laboratory.

All study activities were completed at the NGO study offices in Almaty. Study activities were carried out by NGO staff, from April to August 2010. Data was entered by NGO staff and stored on a secured, web-based application designed to support data capture for research studies. Data were cleaned and analyzed by a JHSPH researcher (MB).

### Analysis

**Risk factor outcomes:** RDSAT statistical software (www.respondentdrivingsampling.org) was used to produce population estimates for behavioral and demographic variables by adjusting for network size and recruitment patterns. Initial analysis included: RDSAT-weighted prevalence of demographics and HIV risk factors. Regression modeling was performed in Stata/SE Version 11. Seeds were dropped from regression analyses, because they were not randomly recruited.

We constructed regression models to determine the association between HIV risk factors and two outcomes: HIV status and UAI with male partners in the last 12 months. Univariate and multivariate logistic regression was used to model the relationship between outcomes and exposures. We hypothesized *a priori* that age, social network size and education were the only confounders in the relationship between the risk factors and the outcomes, and only those variables were used in the multivariate model. No variables were dropped from the models, regardless of statistical significance.

As a sensitivity analysis, we performed analyses in which the outcome was adjusted with weights exported from RDSAT, which is a technique typically used in RDS regression analyses [10], [13]. However, while the prevalence estimates used the RDS weights, the regression analyses presented in this paper do not use weights, because sampling weights do not give correct standard errors in multivariate regressions [14], and there is debate among statisticians on whether RDS weights can be used in multivariate analysis [10], [15]. Instead, we used network size as a confounder in the model to adjust for differences in recruitment due to differences in social network size (i.e. the number of MSM known by the participant), and assessed homophily in results to ensure that homophily did not greatly differ between outcome groups.

## Results

### Prevalence of demographic and risk behaviors

A total of 400 MSM participants were enrolled between April 2010 and August 2010, including the four seeds. All seeds were successful in recruiting other participants. All eligible participants agreed to participate in the interview and HIV screening, and 72.2% of the coupons distributed were returned by recruits seeking to participate. The median number of descendants per seed was 92 (range 83–129). Table 1 shows the adjusted and unadjusted demographic characteristics and table 2 shows access to HIV prevention among the sample of participants. The median age of participants was 28 years (range 18–60, IQR 25–31).More than 47% of participants completed university studies. About 55% self-identified as homosexual and 18.4% as bisexual, though only 21.8% had ever disclosed their sexual orientation to non-MSM friends, family members, or a health care provider. Only about one third of the participants had ever been tested for HIV, and 60% of those participants' last test was more than one year prior to the interview. None of those participants reported having tested positive in their previous HIV tests.

**Table 1:** Crude and adjusted population* estimates of demographic and social characteristics of men who have sex with men (MSM) in Almaty, Kazakhstan (N = 400).

Variable	Sub category	Crude prevalence % (N)	Weighted prevalence % (95% CI)
Age			
	Less than 25 years	22.0 (88)	25.4 (16.9–34.5)
	25–27	26.0 (104)	29.9 (20.8–39.5)
	28–30	24.8 (99)	16.2 (10.6–22.0)
	Over 30	27.2 (109)	28.5 (2.0–38.4)
Marital status			
	Married	15.8 (63)	15.9 (9.7–22.9)
	Live with female partner	4.5 (17)	6.4 (1.8–13.6)
	Live with male partner	24.7 (98)	31.5 (21.4–40.8)
	Single	53.0 (209)	45.4 (36.2–54.9)
Income of more than 200,000 tenge ($1,348) per month		4.5 (18)	6.8 (1.7–14.2)
Had a regular place to live in last 12 months		82.0 (324)	88.6 (83.0–93.9)
Level of education completed			
	General secondary school	16.0 (64)	14.0 (7.8–20.5)
	Specialized secondary school	19.5 (79)	19.5 (12.0–28.9)
	Some university education	20.3 (81)	12.8 (7.6–18.9)
	University	36.0 (142)	47.1 (37.6–56.8)
Sexual orientation			
	Heterosexual	7.7 (31)	10.1 (4.4–17.4)
	Homosexual	64.5 (253)	55.0 (43.8–65.3)
	Bisexual	18.8 (75)	18.4 (12.1–25.5)
	Transgender	5.0 (20)	5.2 (1.4–11.6)
Disclosure of same sex attraction or practices		25.3 (100)	21.8 (12.7–30.7)
	Told non-MSM friends	10.6 (42)	5.4 (2.3–9. 6)
	Told family	8.3 (33)	3.6 (1.4–6.4)
	Told health care provider	2.8 (11)	0.7 (0.2–1.3)

* Raw respondent driven sampling data were adjusted according to the network size and homophily.

**Table 2:** Crude and adjusted population* estimates of HIV prevention practices of men who have sex with men (MSM) in Almaty, Kazakhstan (N = 400).

Variable	Sub category	Crude prevalence % (N)	Weighted prevalence % (95% CI)
Ever tested for HIV		36.0 (141)	33.2 (24.1–42.7)
Conversation with outreach/prevention worker, counselor on how to protect against HIV infection in last 12 months		24.8 (99)	7.6 (4.3–13.3)
Been to a doctor in last 12 months		32.0 (126)	23.1 (15.5–30.8)
Knew correct answer to four HIV questions**		3.2 (13)	3.4 (0.0–7.7)
	Knew anal sex is riskiest type of sex for HIV infection	39.9 (158)	34.4 (25.1–43.9)
	Knew that receptive anal sex is riskiest type of anal sex for HIV infection	11.1 (44)	7.2 (3.4–12.9)
	Knew needles can transmit HIV	44.2 (175)	51.5 (41.3–61.8)
	Knew that water/silicon-based lubricant is the safest lubricant	64.6 (256)	60.6 (50.3–71.1)
Ease of access to free condoms			
	Very easy	10.2 (39)	1.1 (0.6–1.9)
	Somewhat easy	9.5 (36)	4.3 (1.3–9.5)
	Somewhat difficult	12.8 (51)	7.3 (4.2–10.8)
	Very difficult	65.2 (260)	87.0 (81.2–91.6)
Ease of access to water/silicone-based lubricants			
	Very easy	18.0 (69)	17.5 (10.4–26.4)
	Somewhat easy	24.0 (95)	20.5 (13.2–28.6)
	Somewhat difficult	13.5 (53)	10.5 (6.0–15.1)
	Very difficult	30.2 (121)	38.1 (28.8–48.0)

* Raw respondent driven sampling data were adjusted according to the network size and homophily.

** Questions were: “What type of sex puts you most at risk for HIV infection?”; “What type of anal sex puts you most at risk for HIV infection?”; “Can you get HIV from using a needle to inject drugs?”; “What is the safest kind of lubricant for anal sex?”.

Table 3 displays results on prevalence and risk factors for HIV infection among MSM. HIV prevalence in this population is estimated to be 20.2%. These were unknown infections; of those participants testing positive for HIV infection, only 42.7% had ever been tested (CI: 19.3–65.0; unweighted prevalence: 45.3%) and all of those who received their result had been informed the result was negative. Regarding risk behavior, 82.5% of participants reported ever having anal sex without a condom, 69.0% had unprotected anal sex with at least one male partner in the last year, 3.9% had ever injected drugs (most commonly heroin, followed by opium), and 10.9% had used non-prescription and non-injection drugs in the last year. Also, 12.9% of men reported transactional sex (either providing or purchasing sex) in the last 12 months. Homophily between HIV-positives and HIV-negatives was fairly similar (0.07 and 0.44, respectively). The median social network size was 25 for both HIV-positive and HIV-negative participants. Homophily between participants who did and did not have UAI with a male partner in the last 12 months was very similar (0.16 and 0.05, respectively), while the median social network size was 80 and 20, respectively.

**Table 3:** Crude and adjusted population* estimates of HIV risk behavior of men who have sex with men (MSM) in Almaty, Kazakhstan (N = 400).

Variable	Subcategory	Crude prevalence % (N)	Weighted prevalence % (95% CI)
HIV prevalence (by rapid screening test)		13.3 (53)	20.2 (10.6–29.8)
Circumcised		44.5 (177)	34.3 (26.0–43.2)
Binge drinking (five or more drinks in one session) in last 30 days		38.0 (152)	36.3 (26.6–46.0)
Sought partners on internet in last 12 months		38.2 (153)	16.4 (11.0–21.8)
Had concurrent sexual partnerships with at least two people in the last 12 months			
	Yes, male and female partners	16.5 (65.0)	19.3 (12.0–28.5)
	Yes, male partners only	24.0 (94.0)	9.9 (5.7–14.2)
Total number of anal or vaginal sex partners in last 12 months			
	1	25.0 (99)	44.3 (35.2–55.4)
	2–5	44.0 (174)	45.4 (35.8–54.4)
	6 or more	31.0 (122)	10.3 (6.5–13.7)
Number of main partners in the last 12 months (median 1, range 0–12)			
	0–1	83.1 (329)	93.2 (89.4–96.2)
	2 or more	16.9 (67)	6.8 (3.8–10.6)
Number of casual partners in the last 12 months (median 1.5, range 0–250)			
	0–1	50.0 (198)	69.4 (59.9–77.8)
	2 or more	50.0 (198)	30.6 (22.1–40.1)
Unprotected sex with female partners in the last 12 months		18.2 (72)	21.8 (13.3–30.3)
Unprotected sex with male partners in the last 12 months		71.6 (288)	69.0 (59.2–77.5)
Used noninjection drugs in last 12 months		20.5 (81)	10.9 (6.2–16.1)
Ever injected drugs		8.3 (33)	3.9 (1.9–6.1)
Ever had anal sex without a condom		87.5 (345)	82.5 (74.6–89.6)
Unprotected insertive anal sex in last 12 months		57.8 (232)	61.0 (52.6–70.4)
Unprotected receptive anal sex in last 12 months		39.0 (156)	55.5 (45.6–65.4)
STI symptoms in last 12 months		14.2 (55)	6.0 (3.0–9.7)
STI diagnosis in last 12 months		10.4 (41)	3.9 (1.9–6.9)
Transactional sex in the last 12 months		26.0 (100)	12.9 (7.9–19.4)

* Raw respondent driven sampling data were adjusted according to the network size and homophily.

### Risk factors for HIV and unprotected anal intercourse with male partners

Multivariate analysis showed that several risk factors were statistically significantly associated with HIV (table 4). Specifically, knowing the correct answer to four HIV questions was associated with HIV infection (adjusted odds ratio (AOR): 4.56; 95% CI: 1.41–14.78). Receptive UAI in the last 12 months was also associated with HIV infection (AOR: 2.00; 95% CI: 1.04–3.84). Having UAI with a male partner in the last 12 months (table 5) was associated with several factors, including: single marital status (AOR: 0.38; 95% CI: 0.23–0.63); having very difficult access to water- or silicon-based lubricants (AOR: 12.88; 95% CI: 5.65–29.34); self-reported STI symptoms in the last 12 months (AOR: 3.43; 95% CI: 1.41–8.35); transactional sex in the last 12 months (AOR: 3.18; 95% CI: 1.64–8.35); and using non-injection drugs (AOR: 3.10; 95% CI: 1.51–6.36). Further analysis of single marital status found that being single was significantly protective (AOR: 0.22; 95% CI: 0.14–0.36) against having had a main partner in the past 12 months.

**Table 4:** Univariate and multivariate* logistic regression on relationship between risk factors and HIV (N = 396).

Variable	Sub category	Univariate OR (95% CI)	P-value	Multivariate AOR (95% CI)	P-value
Education					
	No university education	1		1	
	Some university education	1.11 (0.62–1.99)	0.73	1.07 (0.59–1.96)	0.82
Age					
	Less than 25 years old	1		1	
	25–27 years old	1.05 (0.43–2.57)	0.91	1.11 (0.45–2.76)	0.82
	28–30 years old	1.56 (0.67–3.65)	0.30	1.62 (0.68–3.84)	0.27
	Over 30 years old	1.27 (0.54–2.99)	0.58	1.24 (0.52–3.00	0.63
Increasing personal network size		1.00 (1.00–1.00)	<0.01	1.00 (1.00–1.00)	<0.01
Increasing income		0.96 (0.78–1.18)	0.70	0.90 (0.71–1.15)	0.46
Marital status					
	Not single	1		1	
	Single	0.99 (0.56–1.77)	0.98	1.12 (0.60–2.09)	0.91
Access to condoms					
	Difficult or easier access to condoms	1		1	
	Very difficult access to free condoms	0.63 (0.35–1.14)	0.13	0.62 (0.34–1.15)	0.27
Access to lubricants					
	Difficult or easier access to lubricants	1		1	
	Very difficult access to lubricants	1.08 (0.58–2.02)	0.80	0.98 (0.51–1.86)	0.69
HIV knowledge**					
	Incorrect answer on at least one HIV question	1		1	
	Knows correct answer to all four HIV questions	4.36 (1.37–13.88)	0.01	4.56 (1.41–14.78)	0.02
Sexual orientation					
	Identifies as heterosexual or bisexual	1		1	
	Self-identifies as “gay/homosexual”	1.66 (0.87–3.17)	0.13	1.64 (0.86–3.15)	0.13
HIV testing					
	Never tested for HIV	1		1	
	Ever tested for HIV	1.59 (0.89–2.86)	0.12	1.51 (0.82–2.80)	0.20
Circumcised					
	No	1		1	
	Yes	0.92 (0.52–1.66)	0.79	0.99 (0.54–1.80)	0.79
STI symptoms					
	Did not report STI symptoms in last 12 months	1		1	
	STI symptoms last 12 months	0.90 (0.38–2.12)	0.81	0.91 (0.38–2.17)	0.81
Health care access					
	Not been to doctor in last 12 months	1	0.56	1	
	Been to doctor in last 12 months	0.82 (0.44–1.56)	0.56	0.77 (0.40–1.48)	0.56
Exchange sex					
	No transactional sex in last 12 months	1		1	
	Transactional sex in the last 12 months	0.85 (0.43–1.69)	0.64	0.86 (0.43–1.72)	0.46
HIV counseling					
	No HIV counseling recently	1		1	
	Met with HIV/STI counselor in last 12 months on male-male sex	1.50 (0.74–3.03)	0.26	1.50 (0.74–3.05)	0.56
Unprotected anal sex with any partner in the last 12 months					
	No	1		1	
	Yes	1.33 (0.64–2.76)	0.45	1.31 (0.63–2.74)	0.60
Unprotected insertive anal sex in last 12 months					
	No	1		1	
	Yes	1.49 (0.81–2.74)	0.20	1.47 (0.80–2.70)	0.21
Unprotected receptive anal sex in last 12 months					
	No	1		1	
	Yes	1.94 (1.02–3.72)	0.04	2.00 (1.04–3.84)	0.04
Had concurrent sexual partnerships with at least two people in the last 12 months					
	Did not have concurrent partnerships	1		1	
	Male and female partners	0.38 (0.13–1.08)	0.07	0.36 (0.12–1.03)	0.08
	Male partners	0.72 (0.34–1.49)	0.37	0.62 (0.28–1.36)	0.23
Use of water- and silicon-based lube in last 12 months					
	Not always use lube during anal sex	1		1	
	Always use lube during anal sex	0.92 (0.50–1.69)	0.79	0.84 (0.44–1.58)	0.42
Binge drinking (five or more drinks in one session) in last 30 days					
	No binge drinking	1		1	
	At least one binge drinking	1.08 (0.59–1.94)	0.81	1.08 (0.60–1.96)	0.81
Noninjection drug use in last 12 months					
	Did not use noninjection drugs	1		1	
	Used noninjection drugs	0.77 (0.36–1.65)	0.50	0.81 (0.37–1.77)	0.44
Injection drug use					
	Never injected drugs	1		1	
	Ever injected drugs	1.85 (0.76–4.50)	0.18	1.98 (0.80–4.88)	0.15
Internet use to look for sexual partners in last 12 months					
	Did not use internet to look for partners	1		1	
	Used internet to look for partners	0.83 (0.45–1.53)	0.55	0.85 (0.46–1.56)	0.55

* Adjusted for age, educational level and social network size in the statistical model.

** Questions were: “What type of sex puts you most at risk for HIV infection?”; “What type of anal sex puts you most at risk for HIV infection?”; “Can you get HIV from using a needle to inject drugs?”; “What is the safest kind of lubricant for anal sex?”.

**Table 5:** Univariate and multivariate* logistic regression on relationship between risk factors and unprotected anal intercourse with male partners in the last 12 months (N = 396).

Variable	Subcategory	Univariate OR (95% CI)	P-value	Multivariate AOR (95% CI)	P-value
Education					
	No university education	1		1	
	Some university education	0.77 (0.49–1.21)	0.26	0.77 (0.49–1.22)	0.27
Age					
	Less than 25 years old	1		1	
	25–27 years old	1.24 (0.67–2.30)	0.50	1.38 (0.73–2.60)	0.32
	28–30 years old	1.68 (0.87–3.21)	0.12	1.89 (0.97–3.69)	0.06
	Over 30 years old	1.26 (0.68–2.33)	0.46	1.55 (0.82–2.96)	0.18
Increasing personal network size		1.00 (1.00–1.00)	0.03	1.00 (1.00–1.00)	0.04
Increasing income		0.92 (0.78–1.08)	0.33	0.88 (0.73–1.06)	0.18
Marital status					
	Not single	1		1	
	Single	0.46 (0.29–0.74)	<0.01	0.38 (0.23–0.64)	<0.01
Access to condoms					
	Difficult or easier access to condoms	1		1	
	Very difficult access to free condoms	0.72 (0.45–1.16)	0.18	0.88 (0.53–1.46)	0.61
Access to lubricants					
	Difficult or easier access to lubricants	1		1	
	Very difficult access to lubricants	9.87 (4.43–21.99)	<0.01	11.08 (4.93–24.91)	<0.01
HIV knowledge**					
	Incorrect answer on at least one HIV question	1		1	
	Knows correct answer to all four HIV questions**	0.86 (0.26–2.88)	0.82	0.92 (0.27–3.18)	0.90
Sexual orientation					
	Identifies as heterosexual or bisexual	1		1	
	Self-identifies as “gay/homosexual”	0.66 (0.41–1.06)	0.09	0.63 (0.39–1.02)	0.06
HIV testing					
	Never tested for HIV	1		1	
	Ever tested for HIV	1.15 (0.72–1.82)	0.56	1.53 (0.69–1.95)	0.77
STI symptoms					
	Did not report STI symptoms in last 12 months	1		1	
	STI symptoms last 12 months	3.93 (1.63–9.47)	<0.01	3.45 (1.42–8.40)	<0.01
Health care access					
	Not been to doctor in last 12 months	1		1	
	Been to doctor in last 12 months	1.07 (0.67–1.72)	0.78	1.05 (0.64–1.71)	0.85
Exchange sex					
	No transactional sex in the last 12 months	1		1	
	Transactional sex in the last 12 months	3.67 (1.91–7.02)	<0.01	3.21 (1.66–6.22)	<0.01
HIV counseling					
	No HIV counseling recently	1		1	
	Met with HIV/STI counselor in last 12 months on male-male sex	0.84 (0.48–1.48)	0.55	0.62 (0.34–1.15)	0.13
Had concurrent sexual partnerships with at least two people in the last 12 months					
	Did not have concurrent partnerships	1		1	
	Male and female partners	0.82 (0.46–1.48)	0.52	0.90 (0.50–1.63)	0.74
	Male partners	0.50 (0.30–0.81)	<0.01	0.39 (0.23–0.66)	<0.01
Use of water- and silicon-based lube in last 12 months					
	Not always use lube during anal sex	1		1	
	Always use lube during anal sex	0.91 (0.56–1.47)	0.64	0.96 (0.57–1.59)	0.79
Binge drinking (five or more drinks in one session) in last 30 days					
	No binge drinking	1		1	
	At least one binge drinking occurrence	1.38 (0.87–2.19)	0.18	1.34 (0.84–2.15)	0.22
Noninjection drug use in last 12 months					
	Did not use noninjection drugs	1		1	
	Used noninjection drugs	3.33 (1.65–6.74)	<0.01	3.10 (1.51–6.36)	<0.02
Injection drug use					
	Never injected drugs	1		1	
	Ever injected drugs	2.30 (0.87–6.13)	0.09	2.03 (0.75–5.48)	0.19
Internet use to look for sexual partners in last 12 months					
	Did not use internet to look for partners	1		1	
	Used internet to look for partners	1.71 (1.06–2.74)	0.03	1.52 (0.93–2.48)	0.08

* Adjusted for age, educational level and social network size in the statistical model.

** Questions were: “What type of sex puts you most at risk for HIV infection?”; “What type of anal sex puts you most at risk for HIV infection?”; “Can you get HIV from using a needle to inject drugs?”; “What is the safest kind of lubricant for anal sex?”.

## Discussion

This study used respondent driven sampling to approximate a random sample of MSM in Almaty, Kazakhstan. The results found a high prevalence of HIV, high levels of risk factors for HIV infection, and low levels of access to health services and HIV prevention programs. In particular, MSM in Almaty tended to report low rates of HIV testing, which is a concern because being tested for HIV is proven to lower risk behavior, as well as provide the first step for entry into HIV care [16].

In multivariate analysis, HIV prevalence was strongly correlated with correctly knowing the answer to four questions about HIV infection, even after adjusting for education, age and social network size. The reason for this finding is uncertain. It could be that people who are exposed to education on risk factors for HIV tend to be people who are already engaged in risk behavior, and self-select for education on HIV. Approximately 40% of the men who tested positive for HIV had previously been tested and, following HCT guidelines, should have received pre- and post-test counseling but may not have altered high risk sexual practices and may have seroconverted in the period between testing. People who are engaged in high-risk sexual networks may also be more likely to be exposed through these networks to the educational interventions existing in Almaty.

Receptive UAI was also associated with HIV infection. This is expected, because unprotected receptive anal sex has a transmission risk probability approximately 18 times higher than vaginal sex [17]. However, other factors that are traditionally associated with HIV infection, such as injecting drug use, not being circumcised, and transactional sex, were not statistically significantly associated with HIV infection in this population. The reasons for this lack of an association are unclear. The HIV epidemic among MSM in Almaty may be a recently developing epidemic, and HIV infections may still be contained within certain sexual networks, while HIV has not yet entered other sexual networks. As the epidemic matures and HIV has had time to spread into more sexual networks of MSM, we expect that the traditional risk factors that affect HIV transmission rates will become statistically significantly associated with HIV infection.

UAI was associated with several other risk factors. Being single (as opposed to married, divorced, or living with a male or female partner) was protective against unprotected anal intercourse. This could be because MSM who are in committed relationships may be having unprotected sex with their main partners, but single men were significantly less likely to have a main partner. There was a strong relationship between very difficult access to lubricants, and a statistically significant association with STI symptoms, trade sex (either giving or receiving favors or money in exchange for sex), and noninjection drug use. Characteristics like low access to lubricants, engaging in transactional sex and noninjection drug use may be proxies for unmeasured variables, such as self-efficacy to use condoms and social support for safe sexual behavior. Given the association with non-injecting drugs observed here, as well as the growing body of evidence pointing to increased sexual risk practices and HIV infection associated with noninjecting drugs, such as methamphetamines [18], further prevention efforts should take drug use into consideration. Moreover, these findings indicate that there is a population of MSM in Almaty with multiple risk factors for HIV infection. While many of these men have not yet been infected, their behaviors indicate that they are at high risk for infection.

Results of the sensitivity analysis (not presented here) indicated that all the risk factors that were statistically significant in unweighted data remained statistically significant in the weighted analysis at p = 0.05. This is a cross-sectional study, and as such, cannot provide evidence of temporality between exposures and outcomes. However, there is a benefit from observing the prevalence of risk factors and associations between risk factors, regardless of temporality. Future studies would benefit from HIV screening in a longitudinal study of MSM, so that the relationship between risk factors and outcomes could be more precisely determined. As a pilot study, this assessment focused on sexual risk, and investigation into drug use among MSM, MSM partnerships, partner behaviors, and networks between MSM groups may be informative for future interventions.

Our enrolled 400 people, which is a modest sample size for an RDS study. This may have resulted in some associations that were due to chance, particularly when a variable had small cell sizes. For example, there was a statistically significant association between HIV knowledge and HIV infection, but only 3.3% of participants in the analysis knew the correct answer to all four questions. Finally, all respondent questionnaires are subject to information biases, such as recall bias and social desirability bias. However, these surveys were conducted anonymously and interviewers were trained in interviewing techniques, in order to minimize these biases.

There are few studies of MSM in Central Asia, and none in the body of peer-reviewed literature that report the use of probability-based sampling. Since RDS is peer-driven, it was able to access a large number of people who may not have otherwise been reached. This study provided one of the first rigorously sampled studies of MSM in Central Asia, which has historically been an understudied population. Furthermore, the involvement of a community-based organization to conduct research and access the most hidden and high risk populations should not be under-estimated. An unusually high proportion of coupons were returned by recruits seeking to participate, which may be partly due to the comfort MSM have with the NGO, and partly due to the fact that only 2 coupons were distributed to participants instead of the traditional 3. The fact participants displayed a variety of unexpected characteristics indicates this was not a biased sample, as would typically be observed with a venue-based sampling method. This study gives an accurate picture of the type and levels of HIV risk behaviors of the diverse population of MSM in Almaty, and their access to HIV prevention materials, so that public health workers can advocate for more HIV prevention interventions and plan effective interventions. In particular, these findings show that there is a group of MSM in Almaty that is engaging in multiple high-risk behaviors, and interventions such as MSM-friendly health care providers and testing sites are needed to prevent the spread of HIV within sexual networks.
